# Improving Cell Recovery: Freezing and Thawing Optimization of Induced Pluripotent Stem Cells

**DOI:** 10.3390/cells11050799

**Published:** 2022-02-24

**Authors:** Markus Uhrig, Fernando Ezquer, Marcelo Ezquer

**Affiliations:** Center for Regenerative Medicine, School of Medicine, Clínica Alemana-Universidad del Desarrollo, Santiago 7610658, Chile; eezquer@udd.cl

**Keywords:** induced pluripotent stem cells, logarithmic cell growth phase, freezing protocol, thawing protocol, post-thaw cell recovery, iPSC storage and transport, preventing osmotic shock, cell aggregates, cell seeding density

## Abstract

Achieving good cell recovery after cryopreservation is an essential process when working with induced pluripotent stem cells (iPSC). Optimized freezing and thawing methods are required for good cell attachment and survival. In this review, we concentrate on these two aspects, freezing and thawing, but also discuss further factors influencing cell recovery such as cell storage and transport. Whenever a problem occurs during the thawing process of iPSC, it is initially not clear what it is caused by, because there are many factors involved that can contribute to insufficient cell recovery. Thawing problems can usually be solved more quickly when a certain order of steps to be taken is followed. Under optimized conditions, iPSC should be ready for further experiments approximately 4–7 days after thawing and seeding. However, if the freezing and thawing protocols are not optimized, this time can increase up to 2–3 weeks, complicating any further experiments. Here, we suggest optimization steps and troubleshooting options for the freezing, thawing, and seeding of iPSC on feeder-free, Matrigel™-coated, cell culture plates whenever iPSC cannot be recovered in sufficient quality. This review applies to two-dimensional (2D) monolayer cell culture and to iPSC, passaged, frozen, and thawed as cell aggregates (clumps). Furthermore, we discuss usually less well-described factors such as the cell growth phase before freezing and the prevention of osmotic shock during thawing.

## 1. Introduction

Induced pluripotent stem cells (iPSC), reprogrammed from somatic cells, offer unprecedented potential for regenerative medicine, drug screening, toxicology, and as cellular disease models [1,2]. Good protocols for cryopreservation and recovery after thawing with little loss in viability are prerequisites for their efficient use. In this review, we focus on the pitfalls common to many of the protocols currently available in the literature. We give an overview of the most important aspects that are important for a successful iPSC recovery such as cryopreservation, storage, transport, and thawing of iPSC, and we also discuss less well-described factors such as the logarithmic cell growth phase before freezing, clone-to-clone variability, and the prevention of osmotic shock during thawing. To complement this information, the protocols for freezing and thawing that we have been using successfully in our laboratory in recent years are available upon request.

## 2. Cryopreservation

### 2.1. General Guidelines for Working with iPSC during the Cryopreservation Process

The absence of microbial contamination in the iPSC culture should be confirmed before freezing. Wearing face masks can prevent the transfer of Mycoplasma from the oral cavity (e.g., Mycoplasma orale) into the cryovials by respiration of the experimenter [3,4,5]. During cryopreservation, it is important to prevent the formation of ice crystals. Ice crystals have been shown to damage cell membranes [6]. Therefore, the tendency of water to form ice crystals must be reduced. Several cryoprotectant agents are used to prevent ice crystal formation such as dimethyl sulfoxide (DMSO), glycerol, propanediol, or methanol. The cryoprotectant agents must be able to cross the cell membrane and penetrate the cells without causing cytotoxicity [7]. Cryoprotectant agents are usually required to be hypertonic to get water out of the cells, which reduces the likelihood of intracellular ice crystal formation. For instance, a 10% DMSO solution in cell culture medium has an osmolarity of approximately 1.4 osm/L [8]. Cells introduced into such a concentrated solution rapidly dehydrate because water leaves the cells to equilibrate the difference in osmotic pressure between the areas inside and outside of the cells. DMSO, in turn, permeates the cells to re-equilibrate. It was discovered that cells are primarily damaged by two factors: intracellular ice formation and cell dehydration [9,10,11]. It has since become clear that preventing these two factors from occurring and their delicate balance are essential for post-thaw cell recovery [12]. Hayashi et al. mentioned that on the one hand, the cooling rate should be slow to avoid intracellular ice crystal formation. On the other hand, the cooling rate should be fast to prevent cell dehydration. Both, intracellular ice formation and cell dehydration, should be balanced in order to assure best cell survival after thawing. Based on these considerations, they assumed three temperature zones for the slow freezing of human iPSC: 1. Dehydration zone, 2. Intracellular ice formation zone (nucleation zone), and 3. Further cooling zone [13]. They performed freeze/thaw experiments using iPSC to measure the cell survival rates at certain cooling rates. These experimental results were used to establish a statistical model for the cell survival rate. Then, this model was used to assess 16,206 three-zone temperature profiles. They determined an optimal three-zone profile and assumed that cell survival is best when an optimal cooling rate is applied for each of these zones [14]. Based on their model, they found that a constant cooling rate did not result in the best cell survival, but that rather a certain cooling rate at the right time during these three zones is the best. They suggested that for optimal cell survival during cryopreservation, it is the best to cool fast in the dehydration zone, followed by slow cooling in the nucleation zone, and again fast in the further cooling zone. This fast-slow-fast pattern is illustrated in Figure 6 in [14].

Human iPSC are more vulnerable to intracellular ice formation than many other human or animal cells [15]. Thus, it is important to strictly control the cooling rate for human iPSC. Ware and colleagues tested different controlled freezing rates for human embryonic stem cells (hESC) and found that a freezing rate within −0.3 and −1.8 °C/min is optimal for cell survival [16]. Li et al. observed a better post-thaw recovery of human iPSC when they were cooled at rates of −1 and −3 °C/min as compared to −10 °C/min. A rate of −1 °C/min is a frequently used freezing rate for iPSC [17]. The best suitable cooling rate is cell type-specific [12]. Oocytes, for example, could be recovered well upon thawing when they were slowly cooled (−0.3 °C/min) to −30 °C, followed by a faster cooling (−50 °C/min) to −150 °C before submerging them into liquid nitrogen [18]. Human oocytes are very susceptible to damage by ice crystals during freezing and thawing because of their large surface area/volume ratio and plasma membrane permeability [19]. Thus, particularly for oocytes, it is important to decrease the possibility of intracellular ice crystal formation [20].

Slow-freezing protocols (controlled-rate freezing) have been shown to result in good cell recovery after thawing [21]. During the freezing process, the cryovials are usually placed in cryocontainers, allowing for slow, controlled-rate freezing in −80 °C freezers, and then transferred to liquid nitrogen tanks or −150 °C freezers for long-term storage. Cells must not be put into liquid nitrogen directly without the controlled-rate freezing step at −80 °C; by doing so, this missing step would harm the cells (an exception to this is the vitrification freezing method, described in Chapter 2.10).

Cryopreservation works best by maintaining cells below the intra- and extracellular glass transition temperatures and staff who wants to store cryopreserved iPSC properly, should ensure that the storage temperature does not rise above these two temperatures [22]. By doing so, all molecular processes cease, and damaging events, e.g., the harmful generation of free radicals, are prevented [23,24]. When thawing cells that had previously been stored at temperatures that were too warm, intracellular ice crystals may be assumed to have formed, possibly damaging the cells mechanically [25,26]. Especially at two temperatures, stressful events may occur that can harm the cells; one is the extracellular glass transition temperature at −123 °C, the other is the intracellular glass transition temperature at −47 °C (−47 °C was reported for Jurkat T cells and may vary for other cell types [22]). The temperature of −123 °C is the extracellular glass transition temperature of DMSO at which the extracellular medium vitrifies. If the frozen cells reach a temperature warmer than −123 °C or warmer than −47 °C, the cells can become stressed, which may reduce viability upon thawing. Thus, cells should not become warmer than −123 °C (or if this happens unintentionally, further warming to temperatures warmer than −47 °C should be prevented because this would inflict another stressful event onto the cells) [22]. Furthermore, Meneghel and colleagues observed particularly high cell mortality when the cells reached temperatures warmer than −25 °C [22]. Warming frozen cells above the extracellular glass transition temperature of −123 °C can be prevented by storing iPSC, for example, in the vapor phase of liquid nitrogen or by storing the cells in −150 °C freezers. The vapor phase in a cryotank has a temperature from approximately −150 to −160 °C, depending on various factors, but can also be warmer than −150 °C or colder than −160 °C, depending on the distance from the level of the liquid nitrogen (Figure 1 in [27]). Depending on the level of liquid nitrogen and the design of the tank, the temperature of the vapor phase can vary, and the level should be monitored continuously so that enough liquid nitrogen remains in the tank (see Supplementary Information for more details). Yuan et al. demonstrated that the long-term storage of iPSC in −80 °C freezers is possible for at least one year without compromising cell viability and pluripotency upon thawing by adding Ficoll 70 to the freezing solution [28].

### 2.2. Effect of Passaging Cells as Aggregates or as Single Cells on Cell Recovery after Thawing

There are mainly two ways of passaging and freezing iPSC: (A) as cell aggregates (clumps) [29] or (B) as single cells [30]. Both ways have advantages and disadvantages. (A) For aggregates, the following advantages/disadvantages are reported in the literature: (1) cell–cell contacts with neighboring cells support cell survival, (2) freezing/thawing of aggregates usually results in a faster recovery compared to single cells because single cells need more time to transition back to form aggregates, (3) the variability in aggregate size leads to a difference in the penetration of the cryoprotectant into the core of the aggregates during the freezing process, which in turn may impact cell viability after thawing. (B) For single cells, the following advantages/disadvantages are described in the literature: (1) better quality control of single cells because cells can be better quantified, resulting in a more consistent recovery time from vial to vial (due to accurate cell counting and viability measurements), (2) single iPSC often need the Rho-associated protein kinase inhibitor (ROCK inhibitor) for survival [31,32]. In this review, we refer to cryopreservation and cell recovery as cell aggregates.

### 2.3. Optimization of Protocol Steps during Cryopreservation

Immediately before freezing iPSC, it is critical to optimize the conditions to achieve successful cell recovery. iPSC clones with a very high proportion of differentiated cells should be discarded because the differentiated cells are an undesirable component of the culture and may induce the differentiation of the (so far undifferentiated) iPSC within colonies. If an acceptable number of spontaneously differentiated cells are present in the iPSC culture, these cells should be removed manually under the microscope before freezing, or by short incubation times with an EDTA-based or another suitable dissociation reagent [33]. The iPSC should be harvested during the logarithmic growth phase (log phase, synonym: exponential phase) before the cells enter the stationary phase and should be frozen subsequently (Table 1); this is described in more detail in the Supplementary Information. iPSC that are frozen during the stationary phase are usually difficult to recover upon thawing [34,35,36].

### 2.4. Logarithmic Cell Growth Phase before Freezing

It has been shown that iPSC recover best after thawing, when they are frozen during the log growth phase [36]. In this phase, cells divide rapidly, which favors a good recovery. For iPSC, it is often not very well-described in the literature when exactly the log phase starts and ends. The best option would be to experimentally determine the beginning and the end of the log phase by measuring cell numbers and generating growth curves. With the goal of reaching high cell numbers (high cell density) before freezing, some experimenters may unintentionally tend to grow the cells beyond the log growth phase and to freeze them subsequently (when the cells have already entered the stationary phase). This, however, leads to poor cell survival after thawing [41]. A straightforward way to reverse this, if the cells are already in the stationary phase, would be to split the cells 1:2–1:4, then let the cells regrow for approximately 2–4 days, and then to freeze them. This strategy takes the cells once again into the log growth phase. After evaluating the current literature, freezing iPSC approximately 2–4 days after passaging proved to be the best means of achieving a successful and fast recovery after thawing [35,36]. Liu and Chen saw the best cell recovery after thawing when iPSC were frozen 2–3 days after passaging. In contrast, they saw poor survival when cells were frozen 5–6 days after passaging [36]. Freezing the cells one day after passaging them, has the disadvantage of the cells possibly still being in the lag phase (not yet in the log growth phase) [35] and that the cell number is still quite small because the cells do not have much time to proliferate within one day. Therefore, we routinely freeze our iPSC 2–4 days after passaging and see good recovery after thawing. It must also be considered that the very first passages of iPSC directly after picking the colonies during reprogramming (approximately passages 1–3, early-passage cells), usually have lower survival rates after cryopreservation, thawing, and plating than those frozen at later passages. Therefore, in our laboratory, we usually add ROCK inhibitor to the cell culture medium when thawing early-passage cells to increase cell survival [42].

### 2.5. Serum-Free Cryopreservation of iPSC

For clinical applications, fetal bovine serum (FBS) cannot be used as a cryopreservation supplement for iPSC due to its risk of disease transmission and possible xenogeneic immune reactions in the transplanted host. Instead, knockout serum replacement (KSR) can be used to replace serum, or serum can be omitted completely without substituting it with KSR [43]. In many laboratories, the freezing medium CryoStor^®^ CS10 (STEMCELL Technologies, Vancouver, BC, Canada) is used. This is a serum-free, animal component-free, defined, and cGMP-manufactured cryopreservation medium containing 10% DMSO. Other serum-free and frequently used freezing media are the CELLBANKER^®^ 2 (Zenogen Pharma, AMSBIO, Cambridge, MA, USA) [44] and STEM-CELLBANKER^®^-GMP grade (Zenogen Pharma, AMSBIO) [45,46]. Liu and Chen dissociated their iPSC with EDTA/PBS (ethylenediaminetetraacetic acid/phosphate-buffered saline) before cryopreservation, and instead of using serum, they froze the cells in 90% Essential 8™ (E8) plus 10% DMSO and did not observe any recovery problems by not adding serum [36]. In our own laboratory, we do not add serum either and have not experienced any problems in cell recovery after thawing as long as other factors were optimized; for example, by ensuring that the cells are in the logarithmic growth phase before freezing, by not disrupting cell aggregates too much during freezing and thawing, and by maintaining a high cell seeding density and adding ROCK inhibitor during thawing [32]. As freezing medium, we use 90% StemFlex™ medium (Thermo Fisher Scientific, Waltham, MA, USA) or 90% mTeSR1™ (STEMCELL Technologies) plus 10% DMSO without adding FBS or KSR. However, it has been described that adding 30% KSR to the freezing medium can improve cell recovery after thawing. The Allen Institute for Cell Science, USA, recommends a freezing medium consisting of 60% mTeSR1™, 30% KSR and 10% DMSO) in one of their protocols. A full cryopreservation protocol is available on the website of the Allen Institute for Cell Science: www.allencell.org/uploads/8/1/9/9/81996008/aics_banking_guidelines_external_v1.3_200211.pdf (accessed on 15 February 2022). Adding 30% KSR supports cell recovery because in their protocol, they dissociate the iPSC with Accutase™ before freezing, which makes the iPSC more vulnerable. The Stem Cell Biobank of the Coriell Institute for Medical Research, USA, recommends a freezing medium consisting of 90% KSR and 10% DMSO (www.coriell.org/0/pdf/NINDS/ipsc/ND41866_Protocol.pdf) (accessed on 15 February 2022). in one of their protocols. Wagner and Welch used two different freezing media—freezing medium A (50% of final volume): 50% DMEM/F12, 50% KSR, and freezing medium B (50% of final volume): 80% DMEM/F12, 20% DMSO. Both media were added to the cells at different times, resulting in a final composition of their cryopreservation medium that was 65% DMEM/F12, 25% KSR, and 10% DMSO [47,48].

### 2.6. Cryopreservation with Serum

A large variability (10–80%) in the ability of iPSC and ESC to attach to coated cell culture plates after thawing has been reported in several laboratories [49,50]. Ha et al. concluded in their publication that adding 5% DMSO + 50% FBS + 10% ethylene glycol to the medium was an optimal cryoprotectant for their slow freezing/rapid thawing protocol of human embryonic stem cells [51]. Fernandes and colleagues could significantly improve cell recovery upon thawing when they added the antioxidant catalase to their FBS containing freezing medium [52]. Some FBS lots (e.g., FBS that is not ESC-qualified) contain growth/differentiation factors which can induce differentiation of iPSC and ESC (e.g., if grown as embryoid bodies) [39,40,53] For this reason, regarding iPSC frozen in a serum-containing freezing solution, it should be considered to remove the serum upon thawing to reduce the risk of unwanted differentiation of the iPSC.

### 2.7. DMSO Concentration in the Freezing Medium

Liu and Chen found that 10–12.5% DMSO in E8 medium provides the best cryopreservation efficiency (Figure 5B in [36]). Furthermore, they found two important facts: 1. EDTA dissociation allowed good survival of human pluripotent stem cells in both passaging and cryopreservation; 2. DMEM/F12 with 10% DMSO was sufficient for cryopreservation when cells were dissociated with EDTA. This is in line with our own observation using 10% DMSO in StemFlex™ medium (Thermo Fisher Scientific) or in mTeSR1™ (STEMCELL Technologies), where we see good cryopreservation efficiency. For cells preserved in a freezing medium containing DMSO, it is generally recommended to wash out or dilute the DMSO immediately post-thaw because DMSO harms the cells at higher concentrations [54].

### 2.8. Different Dissociation Solutions Have a Strong Impact on Cryopreservation and Cell Recovery after Thawing

Membrane adhesion proteins use bivalent ions (2^+^ charged ions), such as calcium- and magnesium ions, to form cell–cell and cell–substrate connections. The membrane adhesion proteins on the cells bind to the 2^+^ charged ions, which act as a link (bridge) between neighboring cells and the substrate (e.g., Matrigel™ coating on plastic dishes). Negatively charged amino acids of the membrane adhesion proteins on the cells bind the 2^+^ charged ions, thus promoting cell adhesion. EDTA is known to be a good chelator of bivalent cations. Thus, EDTA chelates, e.g., calcium- and magnesium ions so that these bivalent ions are no longer available to build the bridge between the cells and the coating on the cell culture dish or between each other.This in turn facilitates cell dissociation. This is reinforced by using a Mg^2+^- and Ca^2+^-free PBS that can be used to wash out Mg^2+^- and Ca^2+^ directly before applying EDTA. Especially for passaging iPSC before cryopreservation, the composition of the dissociation reagent is very important. Beers and colleagues demonstrated that certain combinations of dissociation reagents, cell seeding densities, and the presence or absence of ROCK inhibitor have a strong impact on cell recovery after passaging (Figure 1a in [37]). This also applies to passaging, subsequent cryopreservation, and thawing. They demonstrated that a combination of trypsin or Accutase™ (both *enzymatic*), together with a low cell seeding density and without ROCK inhibitor strongly reduces the survival of iPSC [37]. Other combinations are less problematic: Versene™ (Gibco^®^, Waltham, MA, USA), for example, is a gentle *non-enzymatic* cell dissociation reagent that contains PBS and EDTA. Since it contains no enzymes, it is gentler to the cells; thus, cells can usually be recovered well if combined with a high cell seeding density. Trypsin, in contrast, is more aggressive because it digests the lysine and arginine residues of the carboxy termini of proteins. It digests adhesive cytoskeleton proteins of the cells that anchor the cells to the extracellular matrix. It breaks down cell-to-cell adhesions and cell-to-culture vessel adhesions. In contrast to EDTA, which does not release cell-surface components, trypsin releases large amounts of cell-surface glycoproteins and glycosaminoglycans [55]. Accutase™, which is commercially available, mimics the action of trypsin and collagenase. Accutase™ is a mixture of *enzymes* with proteolytic, collagenolytic, and DNase activity. It is claimed to be gentler than trypsin; however, in combination with a low cell seeding density and without ROCK inhibitor, problems may occur regarding the recovery of pluripotent cells [37,56,57,58].

A suitable dissociation solution should be selected carefully because these solutions have a strong impact on cryopreservation and cell recovery after thawing. *Non-enzymatic* dissociation solutions: The treatment of cell aggregates with (1) non-enzymatic EDTA/PBS produces relatively small cell aggregates during passaging and results in a high survival rate after thawing. Liu and colleagues demonstrated that EDTA-dissociated cells survive better after cryopreservation than cells treated with dispase [36]. (2) To reduce stress for the iPSC and thus increase cell recovery, we opted to use a gentle way of passaging the cells. For this reason, in our laboratory, we use the *enzyme-free* Gentle Cell Dissociation Solution (STEMCELL Technologies, Catalog #07174) in combination with Cell Lifters (STEMCELL Technologies, Catalog #38067), resulting in relatively large cell aggregates during passaging and cryopreservation; consequently, we see good cell recovery after thawing. This is in line with other laboratories which also use the same means for passaging their iPSC [59]. *Enzymatic* dissociation solutions: (1) Dispase, collagenase, TrypLE™, Trypsin, and Accutase™. While treatment with dispase and collagenase results in cell clumps (depending on the incubation time), treatment with Accutase™, TrypLE™, and trypsin results in dissociation into single cells [30,60]. For all enzymatic treatments leading to single iPSC, adding ROCK inhibitor to the medium upon thawing is highly recommended for better cell survival. Beers and colleagues tested different non-enzymatic and enzymatic dissociation solutions for cell passaging and presented a well-illustrated and comprehensible figure in their publication (Figure 1a in [37]). They stressed the importance of maintaining a high cell seeding density for better cell survival, especially in combination with some of their tested dissociation solutions, and observed good cell survival after passaging, e.g., with EDTA [37]. To reduce stress for the iPSC and thus increase cell survival, in our own laboratory, we sometimes use Versene™ (Gibco, catalog number 15040066), a *non-enzymatic* EDTA-containing solution for passaging. For the freezing of our iPSC after passaging with Versene™, we can confirm good cell recovery after thawing which is also reported by Hedges et al. [61]. In our hands, the dissociation of iPSC with EDTA and the subsequent freezing results in smaller cell aggregates upon thawing and plating than by using the Gentle Cell Dissociation Solution in combination with Cell Lifters. In our laboratory, using Versene™ or the Gentle Dissociation Solution™ together with Cell Lifters™ for passaging and the subsequent freezing both result in good cell recovery after thawing.

### 2.9. Economic View on Media, Reagents, and Supplements for the Cryopreservation of iPSC

Working with iPSC can be expensive, but there are ways to reduce costs. The media, supplements, growth factors, and the iPSC themselves are among the most important cost factors. Here, we specifically focus on those factors associated with cryopreservation. High costs are especially caused by using commercially available, integration-free, feeder-free, and xeno-free components. Beers and colleagues established a cost-effective and efficient reprogramming platform, including an optimization of their freezing efficiencies [62]. They worked with a 24–96 well format, which saved costs as compared to the often used six-well format, because a smaller volume of iPSC culture medium was necessary. Instead of expensive commercial dissociation reagents, they used EDTA in PBS. They efficiently cryopreserved iPSC directly on 48-well plates (on-plate cryopreservation of iPSC colonies). They preferred the use of Essential 8 medium (E8, Thermo Fisher Scientific, Gibco #A1517001) over other more expensive media for iPSC. E8 is a xeno-free medium especially formulated for the growth and expansion of human pluripotent stem cells (PSC). E8 was originally developed in the laboratory of stem cell research pioneer James Thomson (University of Wisconsin-Madison). He and his colleagues made their formulation of E8 public so that E8 can be produced by other laboratories, which may reduce costs compared to buying commercially available E8 (Methods section in [49]). The composition of E8 is: DMEM/F12, L-ascorbic acid-2-phosphate magnesium (64 mg/L), sodium selenium (14 µg/L), FGF2 (100 µg/L), insulin (19.4 mg/L), NaHCO_3_ (543 mg/L), and transferrin (10.7 mg/L), TGFβ1(2 µg/L), or NODAL (100 µg/L) [49]. Furthermore, the formulation of mTeSR1 (STEMCELL Technologies, Catalog #38067) is publicly available and can thus be produced by other laboratories at reduced costs (see [63] and Supplementary Table 1 in [64]). For many experiments, such as in vitro experiments, it is not always necessary to use expensive commercial freezing media. Instead, 90% E8 or mTeSR1 medium plus 10% DMSO can be used as a freezing medium provided that other factors are optimized (log growth phase before freezing, using a gentle dissociation reagent such as EDTA for passaging before freezing, etc.). Some laboratories use 90% FBS plus 10% DMSO as a freezing medium for iPSC [65]. A cheaper option would be to reduce the proportion of FBS (e.g., from 90% to 50%) or to use less KSR (e.g., 25% instead of 90%) as an alternative to FBS, which have both been shown to result in good cell recovery upon thawing [47,48,51].

### 2.10. Vitrification

Vitrification (from Latin: *vitrum*, glass) describes the transformation of a substance into a glass-like, non-crystalline, amorphous solid. Vitrification in cryopreservation is a method used to avoid the forming of ice crystals that could harm the cells [66]. During the vitrification process, the forming of ice crystals is prevented by ultra-fast freezing and dehydration of the cells [67]. For vitrification, cells resuspended in a freezing solution in suitable straws or capillaries can be vitrified by submerging them directly in liquid nitrogen (−196 °C) without an intermediary, slow-freezing step in freezing containers. This vitrification procedure transforms the cells, together with the freezing solution, into a non-crystalline glassy phase [68,69]. The following four factors are especially important for cell survival during the vitrification and thawing process: The sample volume, cooling rate, warming rate, and the viscosity [70,71]. The cells are usually resuspended in a very small volume of a freezing solution containing relatively high concentrations of cryoprotectant(s). To avoid the cytotoxic effects of the cryoprotectant, this contact of the cells with the cryoprotectant is kept rather short, directly followed by rapid cooling in liquid nitrogen. The high osmolarity of the freezing solution quickly leads to a dehydration of the cells, and submerging them into liquid nitrogen (−196 °C) rapidly vitrifies the freezing solution together with the cells so that the remaining intracellular water does not have time to form damaging ice crystals. During this process, the cells undergo a transition from 20 °C room temperature to −196 °C in less than two seconds, resulting in very fast cooling rates of −10,000 °C/minute or faster [70,71].

During a successful vitrification process, ice formation is completely prevented. However, for many vitrification protocols, a high concentration of cryoprotectants is needed, and this is usually toxic for sensitive cells such as stem cells and oocytes [18,21,72,73,74,75]. The higher the cooling rate, the lower the cryoprotectant concentration needed for vitrification, and thus the lower the cytotoxicity [76]. Xiaoming He and colleagues developed a quartz microcapillary system to reach ultra-fast cooling rates that lead to vitrification, while using a low, non-toxic concentration of cryoprotectants (2 M intracellular 1,2-propanediol and 0.5 M extracellular trehalose) [77]. Using their quartz microcapillary with an outer diameter of 0.2 mm and a wall thickness of 0.01 mm, they could achieve ultra-fast cooling rates (faster than −100,000 °C/min) that allowed for the use of non-toxic concentrations of cryoprotectants. The very thin wall and diameter of the quartz capillary allowed for such high cooling rates and thus low and non-toxic levels of the cryoprotectants. The boundary heat transfer coefficient, the inner diameter of the quartz microcapillary, and the thermal diffusivity of different materials had a significant effect on the cooling rate (Figure 4 in [77]). Using this vitrification protocol, He et al. reached a survival rate of more than 70% for mouse ESC. These ESC attached well, proliferated normally, and retained the undifferentiated properties of pluripotent cells [77]. Several vitrification methods have been developed for iPSC. High cell survival rates after thawing could be achieved by adherent vitrification or by the CryoLogic vitrification method (CVM) [78,79] In mouse oocytes, it was shown that a rapid warming rate is essential for cell survival. Researchers have concluded that warming rates that are too slow leave time for the forming of small intracellular ice by recrystallization, eventually destroying the cells. If oocyte samples, vitrified in an ethylene glycol-acetamide-Ficoll-sucrose solution, were warmed at a rate of 2950 °C/min, the cell survival rate would be >80%, whereas for those warmed at 139 °C/min, the survival rate would be near 0% [70]. Apart from the positive aspects of the vitrification method, it has to be mentioned that it also comes along with some disadvantages, such as the fact that very small sample volumes and higher cryoprotectant concentrations are necessary, which could lead to cell death if a proper thawing process is not applied [20]. Furthermore, a longer duration of the exposure to these cryoprotectants can be detrimental [80,81], as well as certain temperatures during cooling and warming [82,83]. Gallardo and colleagues stated that despite the ultra-fast cooling and warming rates of vitrification, the procedure as a whole is time-consuming. However, they could shorten the duration of their vitrification protocol by a two-minute exposure to increasingly hypertonic standard cryoprotectant solutions [20].

## 3. Thawing of iPSC

The fast thawing of cryovials containing iPSC is recommended. Slow thawing with slow warming rates can possibly damage the cells by recrystallization or by the prolonged exposure of the cells to high extracellular concentrations of cryoprotectants [84]. For fast thawing, iPSC are typically thawed in a 37 °C water bath, or for clinical-grade iPSC, in a thermoblock or warm bead bath (to reduce contamination risks). After taking the cryovial out of the liquid nitrogen tank, it is recommendable to transport the cells to the water bath in an ice block container in order to prevent a prolonged thawing time, which could reduce viability [67]. Holding the frozen cryovial in the water bath for approximately two minutes is usually sufficient to fast-thaw the cells. Especially if the quality of the frozen iPSC is unknown, it is recommended to add ROCK inhibitor (e.g., Y-27632) to the cell culture medium upon thawing (see Table 2, and described in more detail in the Supplementary Information). The ROCK inhibitor can also be added to the Matrigel™ coating before its polymerization, which has been shown to improve cell attachment after thawing and plating [85]. Pakzad et al. could increase the plating efficiency of human iPSC and human ESC by adding ROCK inhibitor Y-27632 to the Matrigel™. They ruled out possible non-specific effects by adding another ROCK inhibitor (HA-1077/Fasudil, ROCK 2 inhibitor) to the Matrigel™ and also observed a better plating efficiency for this ROCK 2 inhibitor [86].

### 3.1. Plasticware

In our laboratory, we usually coat conventional plastic cell culture dishes (Corning, Falcon, catalog number 353046, 6-well, polystyrene) with Matrigel™ and see good attachment for our iPSC. The Wellcome Sanger Institute also uses these dishes to cultivate their iPSC [93]. Sometimes, we use dishes with a Nunclon™ Delta surface treatment (Thermo Fisher Scientific, Nunc, catalog number 140675, 6-well, treated polystyrene). Nunclon™ Delta is a surface modificationthat makes the polystyrene surface of the culture vessel more hydrophilic. Using the same Matrigel™ coating for these dishes that we also use for the normal plastic dishes, we see a slightly better cell attachment (approximately 10–15% more cells attach to the Nunclon™ Delta surface-treated dishes). These Nunclon™ Delta surface-treated dishes are also used by other laboratories for cultivating iPSC [36,94]. The slightly better attachment may be caused by the hydrophilic surface and/or by the high surface roughness. Nunclon™ Delta had the largest root mean squared (RMS) roughness of about 6 nm compared to the other surface modifications tested; see Figure 2c in [95].

### 3.2. Cell Attachment, Counting, and Viability

Cell attachment and proliferation after thawing are commonly used to evaluate viability. Quantifying post-thaw cell viability is important and may, for example, be performed by using a trypan blue exclusion assay or other methods [96]. While the trypan blue exclusion assay only results in a very rough, unprecise estimation of the number of living and dead cells, there are better options, for example, using an automated cell counting instrument such as the CASY^®^ Cell Counter & Analyzer (OLS OMNI Life Science, Bremen, Germany) [97,98]. This is a highly accurate and precise analyzer for cell lines, primary cells, and all stem cell types, including iPSC and ESC [98,99,100]. The CASY^®^ counter can measure the total cell number. It can also measure the number of viable and dead cells of all possible cell types. No interference with dyes can occur because they are not required for the measurements. Another remarkable feature is its ability to measure cell sizes, their size distribution, cell aggregation, and cell debris [99,101]. Single and aggregated pluripotent stem cells can be measured, making it the ideal device for personalized and regenerative medicine [102]. Reproducible results are displayed in a single graph, taking less than one minute per measurement, with no counting chambers or slides needed. It is GMP/GLP compliant, easy to use, and no sample preparation is necessary. Further recommendable devices that can be used to count cells accurately and precisely are flow cytometers such as the NovoCyte instruments (Agilent, Santa Clara, CA, USA). These are benchtop flow cytometers, with up to five lasers and complex cell analysis capabilities. With an intuitive and straightforward software, iPSC can be quantified easily and rapidly [103]. After freezing, it is good practice to thaw one cryovial of each batch of iPSC clones (frozen on the same day) directly after the cells are frozen (or periodically, at other time points) to assure that the cells attach and recover well, thus confirming a good cryopreservation process. In our laboratory, we observed that iPSC started to attach to Matrigel™-coated cell culture dishes approximately within 30 min after plating (on a feeder-free, Matrigel™ coating; very roughly estimated, 50% of all iPSC colonies should be at least partially attached after 30 min). If they attach significantly later, it indicates that some factors have not been optimized, that a problem has occurred during the freezing/thawing process, or that the iPSC were damaged during storage or transport.

### 3.3. Preventing the Disruption of Cell Aggregates

Throughout the entire process of freezing and thawing, cell aggregates can easily become disrupted, resulting in single cells, which in turn reduces the chances of good cell recovery after thawing. iPSC survival is supported by cell–cell contacts from neighboring cells [37]. When iPSC are singularized, the missing contacts can lead to poor cell survival. (When iPSC are singularized on purpose using, for example, Accutase™, then further protocol steps would be necessary, which are not discussed in this review). The disruption of cell aggregates can happen, for example, through an overly harsh pipetting or by using an unsuitable pipette tip with a lumen that is too small for a proper passaging of the iPSC (e.g., a P1000 µL pipette tip, often blue colored for mechanical micropipettes). Pipetting iPSC through a tip with a lumen that is too small increases the shearing powers, which can in turn lead to the disruption of the cell clumps. Thus, such a pipette tip should not be used, or if so, it should only be used with care, and the possible disruption of cell clumps should be monitored under a microscope. Using 1 mL, 2 mL, 5 mL, or 10 mL serological glass or plastic pipettes mostly prevents the disruption of cell aggregates because these pipettes usually have a larger lumen, which reduces shearing powers. Once the singularization of cells occured, a way to prevent cell death is by adding ROCK inhibitor and plating the cells onto a smaller surface (smaller wells), which forces the cells to become attached in closer proximity to each other, which in turn results in more cell–cell contacts, thus supporting cell survival [31,37,42,58,104,105].

### 3.4. Effects of the ROCK Inhibitor

As already outlined in other chapters of this review, the use of the ROCK inhibitor plays an important role when working with iPSC [37,56,57,58,106]. A final concentration of 10 µM ROCK inhibitor is often added to the cell culture medium during thawing to help the survival of potentially singularized cells [31,37,42,58,104,105]. For better cell survival of iPSC upon thawing, it is recommended and usually sufficient to leave the ROCK inhibitor only for 24 h in the cell culture medium before withdrawing it [42]. It is possible to omit the ROCK inhibitor if all other factors are optimized during freezing, storage, and thawing, if the iPSC are not dissociated to single cells, if the cell seeding density is high enough, and if good quality iPSC are used. Since the quality is sometimes unknown, such as when cells are received from other laboratories or cell banks, it is advisable to add ROCK inhibitor to reduce the risk of sample loss. It was shown by Maldonado and colleagues that the ROCK inhibitor Y-27632 primed human iPSC to selectively differentiate towards the mesendodermal lineage [107]. Y-27632 affected the cell cytoskeleton and cell–cell junction proteins. It induced human iPSC to undergo EMT (epithelial-to-mesenchymal transition)-like changes, which predisposed the cells to differentiate towards the mesendodermal lineage. This was accompanied by a disruption of the actin and E-cadherin organization, resulting in the inhibition of ectodermal differentiation. This has important implications for differentiation protocols. While a prolonged treatment of iPSC with Y-27632 can help to differentiate the cells into cell types of the mesendoderm, it may block their differentiation into ectodermal target cell types such as neurons [107]. Morphologically, the disruption of the actin and E-cadherin organization of the iPSC and ESC (in the presence of Y-27632) is visible in less compact colonies, consisting of cells, which are slightly increased in size and which have a changed shape. This change in morphology is temporary as long as the ROCK inhibitor is on the cells, which is reversed after Y-27632 withdrawal [108]

### 3.5. Preventing Osmotic Shock during Thawing

During thawing, a sudden change in the extracellular osmolarity can induce osmotic stress, which reduces cell viability after thawing [109,110]. Our assumption that iPSC are osmotically shocked during thawing is based on the observation in spermatozoa that are known to be susceptible to osmotic shock during freezing and thawing [111,112,113,114]. In human spermatozoa, osmotic shock during thawing could be reduced by a stepwise reduction of the osmolality in a series of 25 mOsm steps [92]. The European Bank for induced pluripotent stem cells (EBiSC) recommends adding medium *drop by drop* to iPSC in order to minimize osmotic shock during thawing, and they stress in their protocol that this is a crucial step (https://ebisc.org/docs/ebisc/EBiSCProtocolforuseofiPSCv3.pdf (accessed on 15 February 2022). To reduce the likelihood of osmotic shock, after thawing a cryovial with iPSC at 37 °C in a water bath, the cells should first be placed in a 15 mL conical tube, and then the cell culture medium should be added *slowly* in a *dropwise* manner. When the cell culture medium is put into the conical tube first and then the cells are added abruptly, it is more likely that the cells will suffer damage by osmotic shock. The following protocol steps are based on our own experience in the recent years after having worked with iPSC. These steps take some time, but we observed that they result in slightly better cell survival. We observed that at least 15% more cells survived compared to conventional thawing protocols (where the medium was not added stepwise and where no waiting time was provided to slowly equilibrate the osmotic pressure inside and outside of the cells) and we attribute this to a minimized osmotic shock that is accomplished by a stepwise procedure: After thawing one cryovial with iPSC (frozen in 1 mL freezing solution, e.g., 900 µL StemFlex™ or mTeSR1™ medium plus 100 µL DMSO), we first place the cells in a 15 mL conical tube, then we add the following volumes of medium, *slowly* and in a *dropwise* manner—add 1 mL of medium, wait for 5 min (to slowly equilibrate the osmotic pressure inside and outside of the cells), add another 2 mL, wait for 5 min, and finally, add 4 mL and wait for another 5 min. Then, we spin down the cells at 200 g for 2.5 min, aspirate the supernatant, and carefully, without disrupting the cell aggregates too much, resuspend the cell pellet in 2 mL of cell culture medium (mostly in the presence of 10 µM ROCK inhibitor), and seed the cells in one well of a six-well plate previously coated with Matrigel™.

### 3.6. Identifying Problems Causing Insufficient Cell Recovery

**Figure 1:**
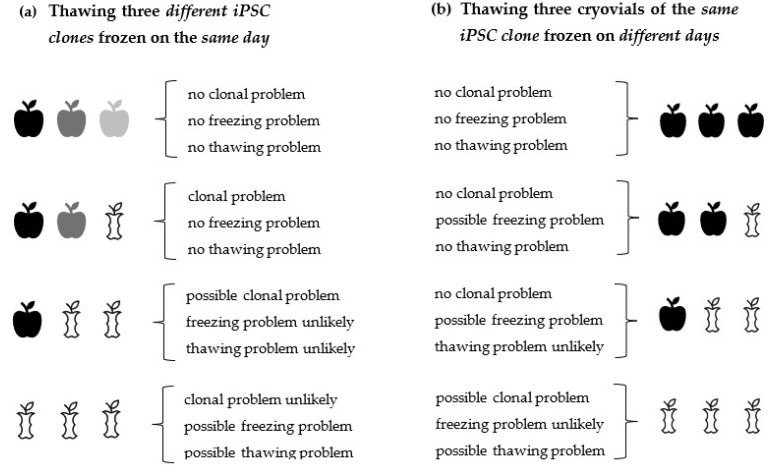
**Decision model that helps to identify problems underlying insufficient cell attachment/recovery upon thawing.** First steps that can be taken to find out if a bad attachment/recovery of iPSC is caused (1) by clonal cell properties, (2) by an unoptimized freezing protocol, or (3) by an unoptimized thawing protocol. After having narrowed down the problem using this decision model, it will be easier to identify further issues more exactly. From this thawing pattern of good/bad cell attachment, one can continue to further identify sources of error systematically. (**a**) By thawing three cryovials containing different iPSC clones, frozen on the same day, information can be obtained about clone-to-clone variability and about the freezing/thawing efficiency. (**b**) By thawing three cryovials of the same iPSC clone, one can get information about this single clone and about the freezing/ thawing efficiency. The term “*freezing problem*” encompasses all steps associated with the entire freezing process, such as being out of the log growth phase before freezing, etc. The term “*thawing problem*” encompasses all steps associated with the entire thawing process, which includes coating problems, etc. Conclusions, given in curved parentheses, are careful approximations of what has actually happened during freezing/thawing. 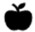 clone 1, 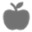 clone 2, 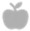 clone 3, 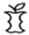 compromised clone.

We suggest taking the following steps whenever iPSC cannot be recovered after thawing. If this is the case, it is often not clear initially what causes this problem. If only one clone is thawed and this clone does not attach properly to the coated cell culture plate after thawing or does not proliferate well, it will not be clear at first glance which of the many possible factors (sub-optimal freezing process, coating, etc.) might be causing this problem. For this reason, we recommend troubleshooting by following a certain order of steps in order to solve the problem more rapidly. Several factors must be considered to find out why iPSC do not show sufficient attachment or survival upon thawing. An effective way to tackle this, is to first find out if the freezing or the thawing procedure is the problem, or if an intrinsic property of the cells themselves, e.g., a clonal effect, is the problem. A suitable strategy to rule out the possibility that not a clonal effect is causing the difference in cell recovery after thawing, is to thaw at least three different iPSC clones in parallel, which have preferably been frozen on the same day (Figure 1a). Further insight into the problem can be gained by thawing three cryovials of an identical iPSC clone, frozen on different days (Figure 1b). Clone-to-clone variability of iPSC occurs occasionally; however, we estimate that freezing or thawing problems take place more frequently than clonal problems, given the fact that during freezing/thawing, many single protocol steps exist, which alone or in combination can lead to insufficient cell recovery after thawing and plating if carried out erroneously or if the protocols are not optimized [115,116].

### 3.7. Supplementary Information

Poor survival of iPSC after thawing and plating can be caused by many factors. We recommend a troubleshooting approach (Supplementary Materials, Figure S1) to identify the underlying problem(s) more quickly. Additionally, we provide information about the shipping of iPSC. iPSC should be shipped according to specific guidelines. A summary of crucial steps can be found in the section *Shipping of iPSC* of the Supplementary Materials and is available online at https://www.mdpi.com/article/10.3390/cells11050799/s1 (accessed on 10 February 2022).

## 4. Conclusions

To make it more likely that iPSC can be recovered successfully after thawing, *both* the freezing *and* thawing protocols should be optimized. The more factors are optimized during cryopreservation and thawing, the higher the chances are of a successful cell recovery after thawing. It also must be taken into consideration that small deviations from the freezing and thawing protocols, which alone do not necessarily cause thawing problems, in combination can add up to a failure in cell recovery after thawing and plating. Whenever there is a problem with cell recovery after the thawing of iPSC, it is initially not clear what it is caused by because there are many factors involved, all of which can contribute to the fact that cells cannot be recovered. Some factors are more important than others for a successful cell recovery, and a general agreement on what is more or less important does not really exist for all these factors. Those factors that we regard as important are compiled in Table 1, Table 2, and summarized in the Supplementary Information in more detail. According to the literature, together with our own hands-on experience, we consider the following factors as essential: (i) freezing of iPSC in the *logarithmic growth phase*, (ii) *handling the cells gently*, *without disrupting the cell aggregates* too much, during freezing and thawing, (iii) *adding ROCK inhibitor* to the cell culture medium during thawing (although it is possible to thaw iPSC without ROCK inhibitor, its use is often recommended, especially if other factors are not optimized, such as working with singularized cells instead of cell aggregates), (iv) maintaining a *high cell seeding density* during thawing, (v) *preventing osmotic shock* during thawing, (vi) using an *optimized coating protocol* (e.g., Matrigel™ prepared according to the manufacturer’s guidelines). Using an optimized thawing procedure is particularly important whenever precious, rare, or expensive iPSC clones are meant to be recovered. Figure 1 summarizes first steps that can be taken to find out if a bad attachment/recovery of iPSC after thawing is caused (1) by clonal cell properties, (2) by an unoptimized freezing protocol, or (3) by an unoptimized thawing protocol. From the resulting thawing pattern of good/bad cell attachment (outlined in Figure 1), one can continue to further identify sources of error systematically. The quality of frozen iPSC is often unknown (for example, when they are delivered from an unknown laboratory/cryobank). Therefore, it is advisable to apply the best possible thawing protocol available and to handle the cells as carefully as possible in order to increase the chance of a good cell recovery. We observed in our laboratory that approximately during the first 1–3 passages directly after reprogramming, a relatively high degree of spontaneous differentiation usually occurs, that the cells are more sensitive and are thus more prone to die. Trying to thaw iPSC, which were frozen at these early passages after reprogramming, can result in low cell recovery after thawing. Extra care must be taken upon thawing of such early-passage cell clones. Alternatively, to circumvent such problems, iPSC can be kept longer in culture, passaged a few more times, and then frozen at later passages (they become more stable after a few passages). In summary, if any thawing problems occur, we suggest the optimization of the essential factors outlined above (factors i–vi of this Conclusion section). Once they are optimized, further possible issues can be troubleshooted, should that become necessary.

## Figures and Tables

**Table 1:** Optimization of protocol steps during freezing.

Factors	Not Optimized	Optimized	Critical Steps	References
Cell growth phase	Cells have already entered the stationary phase.	Freeze cells during the log growth phase, approx. 2–4 days after passaging.	With the goal of reaching a sufficiently high cell number (high confluency), it may happen that the cells are unintentionally grown for too many days and thus have already entered the stationary phase.	[34,35,36]
Cell number	Cell number too low.	Let cells grow up to approx. 70–80% confluency. Freeze iPSC from one well of a six-well plate in one ml of freezing solution (one cryovial).	While the iPSC number is growing, make sure that the cells are still within the log growth phase upon freezing. If necessary, whenever cells have already entered the stationary phase, split 1:2–1:4 and freeze 2–4 days later.	[36,37]
Cell aggregate size	Cell aggregates are disrupted, resulting in single cells or cell aggregates that are too small.	Avoid harsh pipetting. Frequently, a cell aggregate size of 50–200 µm is recommended.	From harvesting until the final steps of freezing, the cell aggregates can be disrupted in many of these steps. Therefore, all steps should be carried out gently *.	[37], STEMCELL Technologies website
Differentiated cells in the iPSC culture	Too many spontaneously differentiated cells appear near or within the iPSC colonies.	Remove differentiated cells manually under the microscope or by short incubation times with an EDTA-based dissociation reagent on a regular basis and directly before freezing.	The number of differentiated cells can vary from clone to clone. iPSC clones which very rapidly differentiate spontaneously should be discarded. Those with an acceptable (low) number of differentiated cells should be manually cleaned on a regular basis (differentiated cells should be removed under a microscope before freezing). Some lots of FBS (e.g., not ESC-qualified FBS) possibly induce differentiation; thus, it may be considered to withdraw FBS after thawing (provided that the freezing solution contained FBS).	[33,38,39,40]

* e.g., by using a gentle dissociation solution for passaging, a gentle handling of the cells, and short centrifugation times (e.g., 2.5 min) at relatively low centrifugal forces (e.g., 200 g).

**Table 2:** Optimization of protocol steps during thawing and plating.

Factors	Not Optimized	Optimized	Critical Steps	References
Coating	Wrong or expired coating substance.	Use good quality Matrigel™ or another suitable coating substance.	Thaw and aliquot Matrigel™ according to SOP on ice *.	[87], Corning and BD Biosciences websites.
Cell number for seeding	Cell number too low, resulting in loss of cell-cell contacts.	Assure high cell density upon seeding.	Try to reach a high cell density **. If necessary, by seeding the given number of cells (e.g., thawed cells from one cryovial) onto a smaller surface (smaller well).	[37,88]
ROCK inhibitor	ROCK inhibitor is missing in the medium.	Add 10 µM ROCK inhibitor.	ROCK inhibitor is helpful for cell attachment and survival, especially if other factors are not optimized (e.g., disrupted cell aggregates, low cell seeding density). If cells are completely singularized, adding ROCK inhibitor is very important for cell survival.	[31,32,37,42,89,90]
Cell aggregate size	Cell aggregates are disrupted, resulting in single cells or cell aggregates that are too small.	Avoid harsh pipetting. Use ROCK inhibitor, especially if aggregates are significantly smaller than approx. 50 µm.	During the whole thawing and seeding process, the cell aggregates can be disrupted. Therefore, all steps should be carried out gently. ***	[37], STEMCELL Technologies website
Osmotic shock	The medium is first poured into a 15 mL conical tube, then all the thawed cells from the cryovial are added suddenly and at once to the medium in the tube.	Put the thawed cells in a 15 mL conical tube first, then add medium *slowly* in a *dropwise* manner.	A sudden change in the osmolarity of the freezing solution around the cells may cause a rapid stream of water across the membranes of the cells. This may stress the cells, making them more prone to die. Avoiding this kind of stress can contribute to better cell survival.	[91,92], European Bank for induced pluripotent stem cells (EBiSC)

* Briefly, thaw Matrigel™ on ice in a refrigerator at 4 °C. Prepare aliquots on ice using pre-chilled tubes and store them at −20 °C or −80 °C. Take Matrigel™ aliquot(s) from the freezer and prepare the coating solution on ice with 4 °C cold medium (DMEM/F12; Matrigel™ will start to form a gel above 10 °C). Pour the coating solution into cell culture dishes and let it gel at room temperature or at 37 °C in an incubator for approximately one hour. ** A low cell density, and consequently a loss of cell–cell contacts, is particularly critical if iPSC are singularized and no ROCK inhibitor is added. *** Gentle handling of cells, short centrifugation time (e.g., 2.5 min) at low relative centrifugal forces (e.g., 200 g).

## Data Availability

Protocols for freezing and thawing of iPSC used in our laboratory are available upon request.

## Supplementary Materials

The following supporting information can be downloaded at: https://www.mdpi.com/article/10.3390/cells11050799/s1, Figure S1: Important steps for cryopreservation, thawing and their optimization options.

## Abbreviations

iPSC	induced pluripotent stem cells
ESC	embryonic stem cells
log phase	logarithmic cell growth phase (synonym: exponential phase)
ROCK inhibitor	Rho-associated, coiled-coil containing protein kinase inhibitor
FBS	fetal bovine serum
DMSO	dimethyl sulfoxide
KSR	knockout serum replacement
DMEM/F12	Dulbecco’s Modified Eagle’s Medium/Ham’s F-12
EDTA	ethylenediaminetetraacetic acid
PBS	phosphate-buffered saline
SOP	standard operating procedure
CVM	CryoLogic vitrification method
