# Supplementary material for: Improving Cell Recovery: Freezing and Thawing Optimization of Induced Pluripotent Stem Cells
Source: Cells. 2022 Feb 24;11(5):799. doi: 10.3390/cells11050799 (PMC8909336; doi:10.3390/cells11050799)
Supplement: Supplementary file 1 [file cells-11-00799-s001.zip › cells-1558315-supplementary.pdf]

Supplementary Information

Troubleshooting approach for iPSC with poor post-thaw survival rates. Poor cell survival after thawing can be caused by many single factors or by a combination of several factors. Since some factors are more important than others, we suggest starting with those that we believe are the most important and common ones. Figure S1 summarizes the most important steps for cryopreservation/thawing and their optimization options. Since all steps, described here, have a strong impact on whether iPSC can be recovered upon thawing, all of them should be optimized. We focus here on passaging and freezing cells as cell aggregates (clumps). Another possibility would be to freeze iPSC as a resuspension of single cells (for this, further protocol steps would be necessary, which are not described here in this review). **Early-passage iPSC:** a less obvious factor that can impede a successful recovery, is that directly after reprogramming of somatic cells into iPSC, during the very first passages (approximately during the first 3 passages), iPSC are more prone to differentiate and the attachment after thawing can be reduced. Therefore, for early-passage cells, it is recommended to use ROCK inhibitor and to seed cells at a high cell density to increase cell survival upon thawing. Furthermore, **clonal effects** must be considered: not all iPSC clones attach and grow equally well. Thus, if problems with attachment and cell growth occur, more than one iPSC clone can be thawed in parallel to discover clone-to-clone variability.

(a) Cryopreservation

Log growth phase

↓

Cell number and aggregate size

↓

Freezing solution

↓

Freezing containers at -80°C

↓

Long term storage in N_2 or -150°C freezers

(b) Thawing

Coating

↓

ROCK inhibitor

↓

Minimizing osmotic shock

↓

Cell aggregate size/seeding density

Figure S1. Important steps for cryopreservation, thawing and their optimization options. All steps of Figure S1 are described in more detail in the text below the Figure legend. **(a) Optimization steps for cryopreservation:** (1) Log growth phase: beginning and end of the log growth phase can be determined experimentally and is approximately 2-4 days after passaging, (2) Cell number (confluency) and aggregate size: 60-80% confluency, 50-200 µm, (3) Freezing solution: 10% DMSO + 90% cell culture medium; FBS or KSR (Knock out serum replacement) can be added alternatively, (4) Freezing containers at -80°C: containers assuring a slow cooling rate of -1°C/min, (5) Long term storage in N_2 or -150°C freezers: it should be monitored that a sufficient level of liquid nitrogen is in the tank assuring a temperature between approximately -150 and -160°C; freezers should be monitored for constant and proper electrical supply. **(b) Optimization steps for thawing:** (1) Coating: Matrigel™ aliquots must be prepared on ice to prevent premature gelling and handling should be done according to the manufacturer's

recommendations, (2) ROCK inhibitor should be added to the cell culture medium at a concentration of 10µM (this is especially important whenever cells are singularized or cell aggregates are very small), (3) Minimizing osmotic shock: freshly thawed iPSC should be put into a conical 15 ml tube first, and then medium should be added to the cells carefully in a dropwise manner, (4) Cell aggregate size/seeding density: 50-200 µm per cell aggregate on average is an often-recommended size at which the aggregates attach well on Matrigel™-coated dishes. For smaller aggregates or singularized cells, it is highly recommended to add ROCK inhibitor. A high cell seeding density may increase cell survival by forming more cell-cell contacts.

The steps of Figure S1 are described here in more detail:

- **Logarithmic cell growth phase (log phase):** 2-4 days after passaging the iPSC as cell aggregates, the cells are mostly within the log growth phase and, according to the literature and our own hands-on experience, this period results in a good cell recovery after thawing. This period may vary slightly, depending on the aggregate size when the iPSC were passaged. Especially, freezing iPSC more than one week after passaging usually results in poor cell recovery. The exact beginning and end of the log phase can be determined by daily cell counting after passaging and generating growth curves.

- **Cell number:** for cell aggregates, iPSC are usually not counted in a Neubauer counting chamber for practical reasons, because for this the aggregates would have to be destroyed. Instead, a cell confluency of approximately 60-80% (surface area of a cell culture well covered with iPSC) is often recommended at which the cells should be harvested and frozen.

- **Cell aggregate size (for freezing):** a cell aggregate size of 50-200 µm is often recommended. It is important not to disrupt the cell aggregates to much by harsh pipetting. A P1000ul pipette tip (often blue colored for mechanical micropipettes) should not be used (or only used with caution while monitoring the clump size under the microscope) during the whole procedure, because the high shearing powers caused by the small lumen of the pipette tip can lead to disruption of the cell clumps. Instead, 1 ml, 2ml, 5ml or 10ml serological plastic or glass pipettes can be used. If though, a pipettor with a P1000µl pipette tip (often blue-colored for mechanical micropipettes) is used, it should be checked under the microscope that the cell aggregates are not getting too small, which means not significantly smaller than 50 µm (this does not apply for protocols where cell aggregates are dissociated into single cells intentionally, for example with Accutase™, and cells are frozen as single cells. For such protocols, further optimization steps would be necessary which are not described here in this review).

- **Freezing solution:** 10% DMSO + 90% cell culture medium is often used as a freezing solution for iPSC. Adding knock out serum replacement (KSR) to the freezing solution is recommended in some publications and by some leading cryobanks to improve cell survival upon thawing. After harvesting the cells, the cell pellet should be carefully resuspended in the freezing solution without disrupting the cell aggregates too much by harsh pipetting. Our standard freezing solution is 10% DMSO + 90% StemFlex™ medium (Thermo Fisher Scientific) or 10% DMSO + 90% mTeSR1™ (STEMCELL Technologies). It is also possible to freeze the iPSC in a freezing solution containing FBS, but it would

be necessary to remove the FBS upon thawing, otherwise the iPSC might differentiate unintentionally.

- **Freezing containers:** a slow freezing protocol in suitable freezing containers is recommended. "Mr. Frosty" (Nalgene) containers are often used. To assure a slow cooling rate of -1°C/min, the container should be filled up with isopropanol (100% isopropyl alcohol) until the fill line indicated on the container (isopropanol should be replaced every fifth use). Alternatively, alcohol-free, cryogenic storage containers can be used, e.g., "Cool Cell" (Corning). Containers with cryovials should be placed in a -80°C freezer for a minimum of 4 hours (usually until 24h), then the cryovials should be removed from the containers and placed in liquid nitrogen or long-term storage freezers (e.g. -150°C freezers).

- **Liquid nitrogen or -150°C long-term storage freezer**. The level of liquid nitrogen in the tank should be monitored regularly and if necessary, filled up to a suitable level. It is recommended to store the iPSC-containing cryotubes in the vapor phase of the liquid nitrogen. Enough liquid nitrogen should always be left in the tank, because not enough liquid nitrogen may lead to an increase in the temperature of the vapor phase above the critical extracellular glass transition temperature of -123°C. -150°C long-term storage freezers should be monitored for proper electrical supply to assure constant cooling.

- **Coating:** Matrigel™ is still the most frequently used coating substance for iPSC. Provided that the Matrigel™ is aliquoted, stored and used according to the manufacturer´s recommendations, iPSC attach and grow well on this coating substance. 1) Preparation and freezing of aliquots: Matrigel™ must be thawed and aliquoted on ice; tubes for aliquots should be pre-chilled at -80 °C and then placed on ice while preparing the aliquots. Otherwise, there is the risk that Matrigel™ gels already in the aliquoted tubes. Then they should be frozen at -20°C or -80°C. 2) Preparation of the coating using the frozen aliquots: aliquots, taken from the -20°C or -80°C freezer, should be placed on ice immediately, without intermittent warming, and ice-cold (4°C) DMEM/F12 should be added. For further details, refer to protocols provided on the website of the manufacturers, for example provided by the company Corning.

- **ROCK inhibitor.** 10μM ROCK inhibitor (Rho-associated coiled-coil containing protein kinase inhibitor) should be added to the cell culture medium to help survival of potentially singularized cells. It is possible to omit ROCK inhibitor, if all other factors are optimized during freezing, storing, and thawing and good quality iPSC are used. Since this is sometimes unknown, for example when cells are received from other laboratories or cell banks, it is advisable to add ROCK inhibitor to reduce the risk of sample loss.

- **Minimizing osmotic shock.** After thawing a cryotube, the iPSC should be put into a conical 15 ml tube first, and then medium should be added to the cells carefully in a dropwise manner. To reduce mechanical force, exerted to the cell clumps in the tubes, the conical tube can be held in a 45° angle to make the process even more gentle, because the medium will gently slide along the tube wall until

it reaches the cells at the bottom of the tube. For precious iPSC samples, we use the following protocol, which takes some time, but results in a slightly better cell recovery after thawing (than conventional thawing protocols): the thawed iPSC should be filled in a 15 ml conical tube first, then the indicated volumes of medium should be added gently to the cells in a dropwise manner: adding 1 ml of medium to the cells, waiting for 5 minutes, adding another 2 ml, waiting for 5 minutes and finally adding 4 ml and waiting for another 5 minutes.

- **Cell aggregate size and seeding density (for thawing)**. Depending on the used dissociation solution and the handling of the iPSC aggregates before cryopreservation, the size of the aggregates resulting after thawing can vary significantly. 50-200 µm per aggregate on average is an often-recommended size at which the aggregates attach well on Matrigel™-coated dishes and at which even the smallest (50 µm) aggregates contain enough cells to support survival by cell-cell contacts. For much smaller aggregates, or even singularized cells, ROCK inhibitor can be added to increase cell survival. Using a P1000ul pipette tip (often blue colored for mechanical micropipettes) is not recommended during the whole procedure, because the high shearing powers caused by the small lumen of the pipette tip can lead to disruption of the cell clumps. Instead, a 1ml, 2ml, 5ml or 10ml serological pipette can be used. A high cell seeding density can also support cell survival. To reach a higher cell seeding density, the iPSC can be thawed and plated on a smaller surface: e.g., iPSC from one cryotube (harvested at 60-80% confluency derived from one well of a 6-well plate), can be seeded in one well of a Matrigel™ coated 6-well plate (instead of seeding them in two or more wells). While one cryotube of high-quality iPSC can usually be thawed in more than one well of a 6-well plate with good cell recovery, this is not necessarily true for iPSC of compromised quality. Thus, for iPSC of unknown or compromised quality, it may sometimes be better trying to recover the iPSC upon thawing by seeding them on a smaller surface (cells will attach in closer proximity to each other, thus cell-cell contacts are more likely to occur which in turn can increase cell survival).

Shipping of iPSC

Cryopreserved iPSC are usually shipped on dry ice (-78,5°C) or in liquid-nitrogen vapor shippers in which the temperature (-150°C or colder) can be monitored. A temperature monitoring system can ensure that the vapor shipper does not become warmer than −130°, for iPSC meant for basic research and −150° for clinical grade iPSC during the shipping process. Traceable data loggers can be used to document the temperature history during transport. Thus, any unexpected warming of the frozen cells above a critical temperature can be tracked back. Upon arrival, it should be checked that the cells can be thawed without problems. Especially for international shipping, sometimes delays may occur at the airport. For this reason, enough dry ice should be placed into the shipping containers to prevent complete evaporation of the dry ice, and thus thawing of the samples during the transport, until the recipient gets the samples. As a general rule of thumb, dry ice sublimates at a rate of 1% per hour in a typical insulated container. Thus, after 3 days of transportation, approximately 30% (of the initial amount) of solid dry ice should be left inside of the container, provided that the shipping container was initially completely filled with dry ice and that the container was large enough. The remaining 30% of

solid dry ice in the shipping container upon arrival is usually enough to keep the cryovials frozen and to recover the iPSC successfully upon arrival. Dry-ice calculators are available on the internet allowing a more precise calculation of container sizes, necessary amount of dry ice and duration of transportation. For longer transportation times (longer than approximately 4 days), using a liquid-nitrogen vapor shipper should be considered, or alternatively, a larger shipping container filled with a larger amount of dry ice. The shipping should be followed by its tracking number and if delays occur at the airport, the officials at the airport and the transportation company should be asked about information of the delivery. Usually, larger airports are equipped with a -20°C or -80°C storage room and, in the case of a transportation delay, the officials at the airport or the transportation company should confirm that the cargo is stored frozen at a suitable temperature.
